# BACE1 activity impairs neuronal glucose oxidation: rescue by beta-hydroxybutyrate and lipoic acid

**DOI:** 10.3389/fncel.2015.00382

**Published:** 2015-10-01

**Authors:** John A. Findlay, David L. Hamilton, Michael L. J. Ashford

**Affiliations:** Division of Cardiovascular and Diabetes Medicine, School of Medicine, Ninewells Hospital and Medical School, University of DundeeDundee, UK

**Keywords:** glucose metabolism, BACE1, amyloid, pyruvate dehydrogenase, mitochondria, alpha lipoic acid

## Abstract

Glucose hypometabolism and impaired mitochondrial function in neurons have been suggested to play early and perhaps causative roles in Alzheimer's disease (AD) pathogenesis. Activity of the aspartic acid protease, beta-site amyloid precursor protein (APP) cleaving enzyme 1 (BACE1), responsible for beta amyloid peptide generation, has recently been demonstrated to modify glucose metabolism. We therefore examined, using a human neuroblastoma (SH-SY5Y) cell line, whether increased BACE1 activity is responsible for a reduction in cellular glucose metabolism. Overexpression of active BACE1, but not a protease-dead mutant BACE1, protein in SH-SY5Y cells reduced glucose oxidation and the basal oxygen consumption rate, which was associated with a compensatory increase in glycolysis. Increased BACE1 activity had no effect on the mitochondrial electron transfer process but was found to diminish substrate delivery to the mitochondria by inhibition of key mitochondrial decarboxylation reaction enzymes. This BACE1 activity-dependent deficit in glucose oxidation was alleviated by the presence of beta hydroxybutyrate or α-lipoic acid. Consequently our data indicate that raised cellular BACE1 activity drives reduced glucose oxidation in a human neuronal cell line through impairments in the activity of specific tricarboxylic acid cycle enzymes. Because this bioenergetic deficit is recoverable by neutraceutical compounds we suggest that such agents, perhaps in conjunction with BACE1 inhibitors, may be an effective therapeutic strategy in the early-stage management or treatment of AD.

## Introduction

Alzheimer's disease (AD) is an age-related neurodegenerative disease, with the vast majority of cases described as sporadic and not currently linked with a specific gene defect. There are approximately 44 million people living with AD in the world, and this number is predicted to increase dramatically over the next three decades, severely impacting on healthcare systems and the socio-economic environment. AD encompasses large-scale neuronal loss and progressive memory and other cognitive decline culminating in severely impaired daily living. Indeed, there is a strong correlation between synapse loss and cognitive deterioration (DeKosky et al., 1996; Coleman and Yao, 2003). The neuronal cell death follows a characteristic spatial and temporal pattern; with signs of the disease initially presenting in layer II neurons of the entorhinal cortex (EC) and spreading through the hippocampus (Gómez-Isla et al., 1996). Selective atrophy and regional hypometabolism have also been shown to occur in the EC through the use of functional imaging (Masdeu et al., 2005). Allied to this, loss of cholinergic neurons and reduced cholinergic system enzyme activities in the basal forebrain have been proposed to drive early cognitive decline (DeKosky et al., 1992; Perry et al., 1992; Geula et al., 2008). Presently, we have a limited understanding of the molecular basis for these early events in sporadic AD etiology. Recent proteomic analyses have however highlighted a number of cellular processes, which become compromised. These include the pathways of metabolism and energy production, oxidative stress production and defense culminating in DNA and protein modifications impacting on cytoskeletal functioning (Sowell et al., 2009; Korolainen et al., 2010). All of these changes culminate in alterations in synaptic communication and precipitate memory loss (Forero et al., 2006). It has been hypothesized that a major driver of these changes is the aberrant folding and phosphorylation of the microtubule associated protein tau and aggregation of β-amyloid (Aβ) peptides (Kawaja, 2005). While the progression of AD is closely associated with dysfunctional tau protein and cholinergic deficits, it is generally observed by biomarker and other analyses that changes in the hallmark amyloid plaque pathology precedes these other pathogenic drivers (Shankar et al., 2009; Jack et al., 2013a,b; Knopman et al., 2013; Martiskainen et al., 2015). There has also been increasing interest in the role of brain mitochondrial dysfunction and reduced metabolic activity (Yao et al., 2009; Swerdlow et al., 2014; Wilkins et al., 2014).

It has long been recognized that glucose is the predominant substrate utilized by the adult brain under physiological conditions (Bouzier-Sore et al., 2006). Indeed, while only constituting 2% to body weight, the brain consumes around 20 and 25% of the total body oxygen consumption and glucose respectively, (McKenna et al., 2006). Furthermore, the respiratory quotient of the brain is almost exactly 1, indicating near universal carbohydrate metabolism (Sokoloff, 1999). Although glucose is the dominant substrate for brain metabolic activity, alternative substrates, such as glycogen and amino acids can play a role in central metabolism. However, due to limited supply and storage capacity, this is thought to be relatively minor under physiological conditions (Henderson et al., 2009). Glucose metabolism in the brain is tightly coupled to the generation of intracellular adenosine triphosphate (ATP), largely to support neurotransmitter production and release. Consequently, this reliance on glucose coupled with a high-energy consumption makes the brain, and synaptic transmission in particular, vulnerable to events, which lead to diminished metabolism.

Brain hypometabolism is a universal change observed during Alzheimer's disease (AD) progression and recently, it has been proposed to have a major causative role in AD pathogenesis (Blass, 2001; Costantini et al., 2008). Through the use of neuroimaging techniques, impaired brain glucose metabolism has been demonstrated to occur: before atrophy in autosomal AD cases, during the progression toward non-familial AD and in individuals at high risk of AD (i.e., APOE ε4 carriers) prior to symptom manifestation (Small et al., 1995; Johnson et al., 2001; Reiman et al., 2004; Mosconi et al., 2009a,b). This reduced brain glucose metabolism has been postulated to result from impaired mitochondrial functioning (Lustbader et al., 2004; Beal, 2005; Bubber et al., 2005), with similar changes observed in a variety of transgenic mouse models of AD (von Kienlin et al., 2005; Yao et al., 2009, 2011; Nilsen et al., 2014). Recent evidence suggests a putative link between neuronal glucose metabolism and the presence of Aβ peptides, considered by many to be the primary pathogenic driver of AD. Aβ peptides derive from increased cleavage of amyloid precursor protein (APP), which is ubiquitously expressed in tissues, with highest levels in the brain. APP is cleaved by two competing enzyme processes; an alpha secretase pathway, resulting in the soluble cleaved product sAPPα and no Aβ production and a beta secretase pathway, which releases sAPPβ and following additional cleavage of the remaining protein by γ-secretase, releases Aβ. Aβ accumulates in the mitochondria of AD patients and AD mouse models, prior to the appearance of amyloid deposits (Hirai et al., 2001; Devi et al., 2006; Manczak et al., 2006). Indeed, exogenous Aβ has been reported to decrease mitochondrial respiratory chain function and the activity of various mitochondrial dehydrogenase enzymes (Shearman et al., 1994; Casley et al., 2002a; Pereira et al., 2004; Caspersen et al., 2005), indicative of an Aβ-mediated bioenergetic deficit in cells.

Additionally, it has been shown that the hypometabolic state associated with AD may be driven, at least in part, by a region-specific shift in neuronal metabolism toward aerobic glycolysis (reduced oxidative metabolism in the presence of adequate oxygen supply; Vaishnavi et al., 2010). This switch in glucose metabolism was shown to closely correlate with Aβ deposition and later vulnerability to cell death during progression toward AD (Vlassenko et al., 2010). These findings clearly implicate the modulation of APP processing as a driver of the altered metabolic state observed in early, pre-AD states. A key enzyme driving excess Aβ production, as observed in AD, is the aspartyl protease, β-site APP cleaving enzyme 1 (BACE1). BACE1 was initially characterized as the enzyme controlling the rate-limiting step in Aβ generation (Hussain et al., 1999; Sinha et al., 1999; Vassar et al., 1999). More recently however, it has also been proposed to play a role in glucose metabolism with whole body knock out of BACE1 resulting in improved insulin sensitivity and glucose homeostasis (Meakin et al., 2012). Furthermore, genetic and pharmacological manipulation of APP processing can directly alter glucose uptake and metabolism in the C2C12 skeletal muscle cell line (Hamilton et al., 2014). Importantly, BACE1 is a stress-sensitive protease, with oxidative, hypoxic, inflammatory, and metabolic stress (all associated with AD initiation and/or progression) demonstrated to increase BACE1 levels and activity, causing APP processing to shift from the physiologically predominant alpha-secretase, to the beta-secretase, pathway, and increasing Aβ levels. Indeed, recent work has demonstrated a role for oxidative, lipid and metabolic stressors in regulating BACE1 gene and protein expression as well as activity (Puglielli et al., 2003; Velliquette et al., 2005; Zhang et al., 2007; O'Connor et al., 2008; Guglielmotto et al., 2009). Furthermore, alterations in its expression result from changes in micro RNA (miR) regulation of the 5′ untranslated region (UTR) of BACE1 have been demonstrated in sporadic AD cases (Nelson and Wang, 2010; Fang et al., 2012; Lei et al., 2015). Consequently, chronic stress events and altered translational regulation may in turn culminate in the consistent reports that BACE1 mRNA, protein and activity are elevated in AD brains (Fukumoto et al., 2002; Holsinger et al., 2002; Tyler et al., 2002; Yang et al., 2003; Li et al., 2004; Harada et al., 2006; Zhao et al., 2007).

Given that Aβ directly impairs mitochondrial enzyme function and that AD is associated with impaired glucose metabolism we hypothesized that manipulating APP processing through BACE1 overexpression in SH-SY5Y neuroblastoma cells would phenocopy the defects in glucose metabolism at the cellular level, allowing us to explore in more detail this initial and potentially causative change in AD progression.

## Materials and methods

### Cell culture

SH-SY5Y cells were cultured under aseptic conditions and maintained in a humidified atmosphere of 95% air and 5% CO_2_. Cells were maintained in Dulbecco's Modified Eagle's Medium (DMEM) F-12 media (Gibco Life Technologies, Paisley, UK) supplemented with 10% fetal bovine serum (Sera Laboratories, West Sussex, UK), 4 mM L-glutamine (Gibco) and 2% Penicillin streptomycin (100 units/ml; Gibco). SH-SY5Y cells were transfected with 12 μg DNA of pcDNA3.1 containing empty vector (SH-SY5Y_EV_), full-length human BACE1 (SH-SY5Y_B1_), or BACE1 active site mutant (SH-SY5Y_mB1_) using Lipofectamine 2000 (Invitrogen Life Technologies, Paisley, UK). Stable cells were selected and lines maintained using 1 and 0.5 mg/ml G418 sulfate respectively, (Sigma-Aldrich, Gillingham, UK) as selection antibiotic. A minimum of two independently generated stable cell lines, with concurrently produced EV controls, were used for these studies. Prior to some glucose oxidation assays, cells were treated overnight, as indicated, with the pyruvate dehydrogenase inhibitor, dichloroacetate (DCA; Sigma-Aldrich) or growth media supplemented with α-lipoic acid (Sigma-Aldrich) for 48 h.

### Cloning

Full length myc-his tagged human BACE1 in pcDNA3.1 was obtained from GlaxoSmithKline (GSK; Harlow, UK) and mBACE1 (a kind gift from Professor Wolfe (Brigham and Women's Hospital, Boston)) was sub-cloned into pcDNA3.1.

### Immunoblotting and gene expression

Protein isolation and immunoblotting procedures were as described previously (Mirshamsi et al., 2004). For relative quantification of APP cleavage by alpha-secretase (sAPPα) vs. beta-secretase (sAPPβ) pathways, cells were incubated for between 20 and 24 h in Optimem (Gibco), and media concentrated (using 30 kDa Amicon Ultra 15 ml filters; Merck Millipore, Livingston, UK) by centrifugation (4000 × g) and subjected to SDS-PAGE with amounts presented relative to total protein. Protein antibodies used were: anti-Actin (Sigma-Aldrich; 1:5000), anti-APP (Ab54, GSK; 1:4000), anti-sAPPα (Cambridge Bioscience, Cambridge, UK; 1:1000), anti-sAPPβ (GSK; 1:1000), anti-BACE1 (Sigma-Aldrich; 1:1000), anti-BAD (New England Biolabs, Hitchin, UK; 1:1000), with anti-total PDH (1 mg/ml) and anti-pPDHe1α (1 mg/ml) from Drug Discovery Unit, University of Dundee.

### Glucose oxidation assay

SH-SY5Y_EV_, SH-SY5Y_B1_, or SH-SY5Y_mB1_ cells were plated into 6-well cell culture plates and any pre-treatments carried out as described above and in the Results Section. To begin the assay, cells were washed twice with Hepes-buffered saline (HBS (in mM); 140 NaCl, 20 Hepes, 5 KCl, 2.5 MgSO_4_, and 1 CaCl_2_, pH 7.4) and incubated in HBS containing 2.5 mM glucose and 74 kBq/ml D-[U-^14^C]glucose (PerkinElmer) along with any relevant inhibitors or treatments indicated in the Results Section for 3 h at 37°C. Media were transferred and ^14^CO_2_ liberated by acidification with 60% perchloric acid, trapped by Whatman (GF/B) filter paper discs pre-soaked with 1 M KOH and radioactivity quantified by liquid-scintillation counting. Cells from the assay were washed twice with ice-cold 0.9% NaCl and lysed with 1 ml of 50 mM NaOH, and the radioactivity contained within the lysate quantified by liquid-scintillation counting, which served as a measure of the ^14^C incorporation into the cell during the assay period. Total protein content was determined in the lysate via the Bradford method and used to normalize glucose incorporation and oxidation rates for each sample.

### Cellular respiration

The Seahorse Extracellular Flux Analyser utilizes solid sensors that simultaneously monitor the oxygenation and pH of the media. The rate of oxygen consumption (OCR) and extracellular acidification rates (ECAR) can therefore be assessed in near real time allowing for high resolution changes in a range of metabolic parameters. SH-SY5Y_EV_, SH-SY5Y_B1_, or SH-SY5Y_mB1_ cell monolayers were seeded into XF 24-well culture microplates (Seahorse Bioscience, Copenhagen, Denmark) the day prior to treatment or assay as indicated in the Results Section. Optimal cell number was determined following a cell titration assay taking into account oxygen consumption rate (OCR) and extracellular acidification rate (ECAR), oxygen tension values and the appearance of the cell monolayers and was determined to be 40,000 cells. On the day of the assay, cells were placed in unbuffered DMEM with relevant inhibitors/treatments as indicated and placed in a non-CO_2_ incubator for 1 h (to de-gas solutions) prior to assay initiation. Standard 3 min mix, 2 min wait, and 3 min measure cycles were used; with five baseline measurements taken before, and a subsequent three measurements acquired following, drug additions.

### Enzyme activity assays

Activity assays for pyruvate dehydrogenase (PDH; Abcam, Cambridge, UK), α-ketoglutarate dehydrogenase (α-KGDH; Antibodies Online, Aachen, Germany), isocitrate dehydrogenase (IDH; Sigma-Aldrich), and fumarase (Abcam) were performed according to the manufacturer's instructions.

## Results

### Overexpression of BACE1 increases amyloidogenic APP processing and suppresses glucose oxidation

**Figure 1:**
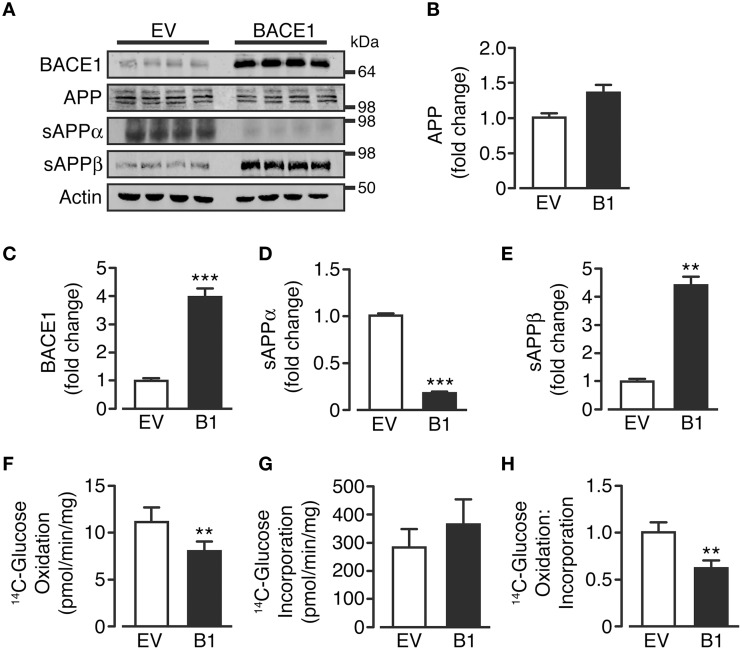
**BACE1 overexpression alters APP processing and glucose oxidation in SH-SY5Y cells**. **(A)** Representative immunoblots showing stable overexpression of BACE1 and resultant changes in cellular APP and sAPPα and sAPPβ shed into the culture media, compared to empty vector (EV) treated controls. **(B–E)** SH-SY5Y BACE1 overexpression did not change APP protein levels (**B**; *n* = 4), increased total BACE1 protein levels (**C**; *n* = 7) and induced a shift in APP processing, with a reduction in sAPPα (**D**; *n* = 4) and an increase in sAPPβ (**E**; *n* = 4). (**F–H)** SH-SY5Y BACE1 overexpression reduced ^14^C-glucose oxidation rate (**F**; *n* = 7), with no reduction in ^14^C-glucose incorporation rate (**G**; *n* = 9), giving an overall reduction in the ratio of ^14^C-glucose oxidation to incorporation (**H**; *n* = 7). Values are means ± SEM. ^**^p < 0.01; ^***^p < 0.001.

Control, empty vector-treated (SH-SY5Y_EV_) and BACE1 overexpressing (SH-SY5Y_B1_) cells displayed equivalent APP protein levels, showing the three predominant APP transcript protein isoforms present in neurons (Huang and Jiang, 2011; Figures 1A,B). As expected, SH-SY5Y_B1_ cells exhibited significantly higher BACE1 protein levels (Figures 1A,C), which resulted in altered APP cleavage; promoting a substantial shift from non-amyloidogenic to amyloidogenic processing (as denoted by increased sAPPβ and decreased sAPPα levels (Figures 1A,D,E). Overexpression of BACE1 resulted in a marked reduction in the rate of ^14^C-glucose oxidation, compared to SH-SY5Y_EV_ cells (Figure 1F), in agreement with recent findings in C_2_C_12_ skeletal muscle cells (Hamilton et al., 2014). The decrease in glucose oxidation is not due to impaired glucose incorporation into the cell (Figure 1G), and suggests that increased BACE1 protein levels and activity cause a fundamental change in the ability of SH-SY5Y cells to oxidize glucose (Figure 1H).

**Figure 2:**
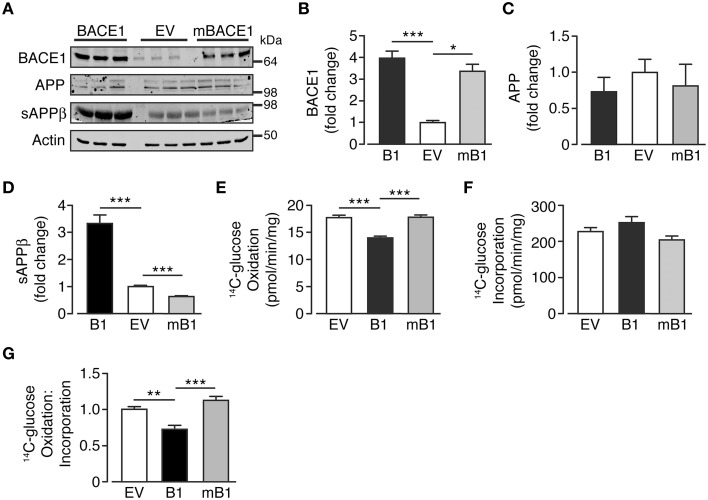
**Glucose oxidation in SH-SY5Y cells in not impaired following overexpression of secretase-dead BACE1 protein**. **(A)** Representative immunoblots showing stable overexpression of wild type BACE1 and a secretase-dead BACE1 mutant (mBACE1) and resultant changes in cellular APP and sAPPβ shed into the culture media. **(B–D)** SH-SY5Y mBACE1 overexpression increased total BACE1 to levels equivalent to BACE1 overexpressed cells and ~3-4X greater than empty vector (EV) controls (**B**; *n* = 5), with no effect on APP levels (**C**; *n* = 3), but in contrast to BACE1 overexpression, mBACE1 reduced sAPPβ to levels below that of the EV controls (**D**; *n* = 6–10). **(E–G)** SH-SY5Y mBACE1 overexpression had no effect on ^14^C-glucose oxidation compared to EV controls, in contrast to the reduction in ^14^C-glucose oxidation observed in BACE1 overexpressing cells (**E**; *n* = 5), with mBACE1 having no effect on ^14^C-glucose incorporation (**F**; *n* = 5), resulting in the ratio of ^14^C-glucose oxidation to incorporation being unchanged in mBACE1 cells (**G**; *n* = 5). Values are means ± SEM. ^*^p < 0.05; ^**^p < 0.01; ^***^p < 0.001.

In an attempt to discern whether the changes in glucose oxidation rate were dependent upon the secretase activity of BACE1, a concomitant group of cells that overexpress a mutant form of BACE1, devoid of secretase activity (SH-SY5Y_mB1_; Mowrer and Wolfe, 2008) were examined. BACE1 protein levels in SH-SY5Y_mB1_ cells were raised to a similar extent to that observed for SH-SY5Y_B1_ cells, with no alteration in APP protein levels (Figures 2A–C). Interestingly, the overexpression of the secretase-dead BACE1 mutant exerted a dominant negative effect over APP processing with reduced sAPPβ release, compared with SH-SY5Y_B1_ and SH-SY5Y_EV_ cells, into the culture media (Figures 2A,D). The reduced β-secretase cleavage of APP in the presence of mBACE1 was also mirrored in its effect on glucose oxidation rate, with no reduction in glucose oxidation concomitant with comparable glucose incorporation between cell types (Figures 2E,F), indicating that increased BACE1 activity is required to depress glucose oxidation in SH-SY5Y cells (Figure 2G).

### Chronic elevation of BACE1 stimulates aerobic glycolysis in SH-SY5Y cells

**Figure 3:**
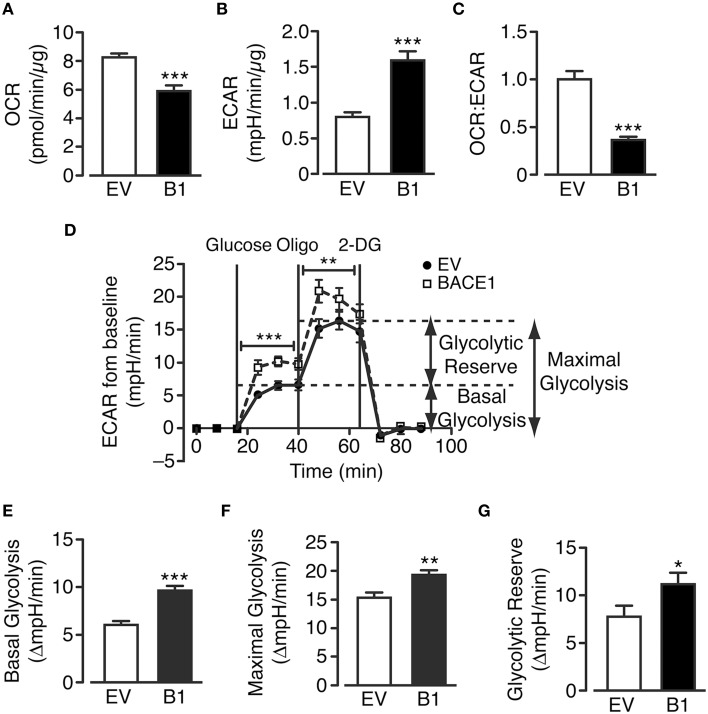
**BACE1 overexpression alters glucose metabolism in SH-SY5Y cells**. **(A–C)** BACE1 overexpression reduces oxygen consumption rate (OCR) **(A**) and increases the extracellular acidification rate (ECAR) **(B)** resulting in a reduced OCR:ECAR ratio **(C)**, compared to EV cells (*n* = 7–12). **(D)** Glycolysis stress test temporal profile for EV and BACE1 SH-SY5Y cells showing the effects on ECAR of the sequential addition of 2.5 mM glucose, 1 μM oligomycin (Oligo) and 25 mM 2-deoxyglucose (2DG). **(E–G)** BACE1 overexpression increased basal glycolysis **(E)**, maximal glycolysis **(F)** and glycolytic reserve **(G)** compared to EV-treated cells (*n* = 18–21). Values are means ± SEM. ^*^p < 0.05; ^**^p < 0.01; ^***^p < 0.001.

The cellular pathways that facilitate glucose metabolism are glycolysis (and the pentose phosphate pathway), which occurs in the cytoplasm and the TCA cycle and oxidative phosphorylation, which are present in the matrix and inner mitochondrial membrane, respectively. Actively respiring mitochondria consume oxygen and therefore oxygen consumption rate (OCR) can be taken as a measure of substrate flux through the oxidative phosphorylation pathway while extracellular acidification rate (ECAR) reflects the release of lactate (lactic acid) converted from pyruvate following glycolysis. SH-SY5Y_B1_ cells exhibit decreased OCR concurrent with increased ECAR in comparison to SH-SY5Y_EV_ cells (Figures 3A,B). Taken together these changes reflect a robust shift between these metabolic processes as shown by the change in the ratio of oxidative phosphorylation to glycolytic metabolism in cells with increased BACE1 activity (Figure 3C).

To further investigate these changes, a glycolysis stress test (which assesses basal glycolysis, glycolytic capacity and glycolytic reserve) was performed (Figure 3D). SH-SY5Y_EV_ and SH-SY5Y_B1_ cells were incubated in media containing zero glucose, 2.5 mM pyruvate and 4 mM L-glutamine for 100 min, following which stimulation of basal glycolysis was achieved by the injection of 2.5 mM glucose into the assay media. The increase in basal ECAR was significantly higher in SH-SY5Y_B1_ cells, compared to control cells, indicating that the raised BACE1 activity augmented aerobic glycolysis (Figure 3E). Inhibition of F_1_F_0_ ATP synthase by oligomycin leaves cells wholly reliant on glycolysis for ATP generation (termed maximal glycolysis). This rate was also significantly increased in SH-SY5Y_B1_, compared to control, cells following BACE1 overexpression (Figure 3F). Finally, the cellular glycolytic reserve is given by the difference between basal and maximal glycolysis, with a significantly higher glycolytic reserve observed in the SH-SY5Y_B1_ cells (Figure 3G). Collectively, these data indicate that raised BACE1 activity depresses glucose oxidation in SH-SY5Y cells and, as a result, there is a compensatory increase in glucose metabolism through aerobic glycolysis.

### Mitochondrial efficiency is unaltered by raised BACE1 activity in SH-SY5Y cells

**Figure 4:**
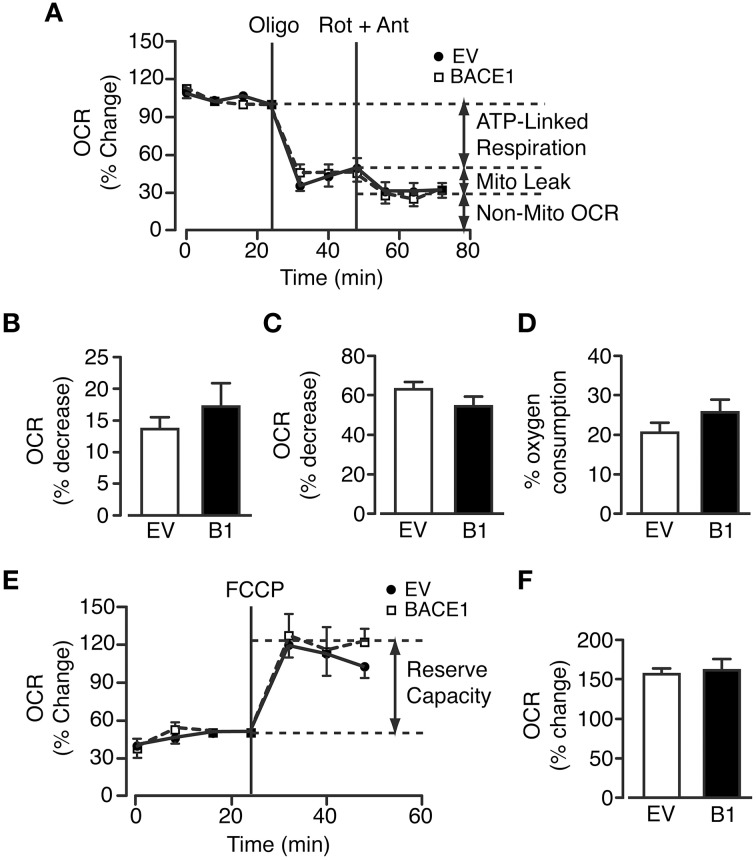
**BACE1 overexpression has no effect on ATP-linked, maximal or leak mitochondrial oxygen consumption**. **(A)** Modified mitochondrial stress temporal profile for EV and BACE1 SH-SY5Y cells showing the effects on the percentage change in normalized OCR of the sequential addition of 1 μM oligomycin (oligo) and 2 μM rotenone and antimycin (Rot + Ant). **(B–D)** BACE1 overexpression had no effect on ATP-linked OCR **(B)**, on the OCR required to maintain the mitochondrial leak **(C)** or the non-mitochondrial OCR **(D)** compared to EV cells (*n* = 8). **(E)** Temporal profile showing the normalized baseline OCR for EV and BACE1 cells and the maximal OCR (reserve capacity) attained following addition of 0.2 μM carboyl cyanide-p-trifluoromethoxyphenylhydrazone (FCCP). **(F)** BACE1 overexpression has no effect on the percentage OCR increase following FCCP addition compared to EV cells (*n* = 9–10). Values are means ± SEM.

The marked reduction in oxidative phosphorylation, concurrent with increased glycolytic metabolism in SH-SY5Y_B1_ cells, indicated that increased BACE1 activity reduced substrate delivery to the mitochondria and/or impaired mitochondrial function. To test mitochondrial efficiency in these cells, a modified Mitochondrial Stress Test (Seahorse Bioscience) protocol was utilized. As previously reported, a limited maximal respiration rate is achievable in undifferentiated SH-SY5Y cells (Xun et al., 2012) in the absence of pyruvate in the assay media. Consequently, we therefore performed a split assay to investigate the effects of mitochondrial metabolism perturbation: the first part measuring the proportion of respiration dedicated to ATP generation (ATP synthase inhibition with oligomycin), the maintenance of mitochondrial leak (a combination of rotenone (complex I inhibition) and antimycin A (complex III inhibition) to minimize mitochondrial respiration) and the relative contribution of non-mitochondrial OCR (Figure 4A). However, increased BACE1 activity in SH-SY5Y cells had no effect on any of these oxidative parameters (Figures 4B–D). The second part encompasses a test of the cellular reserve capacity via the induction of maximal respiration through addition of the proton ionophore FCCP. This drug collapses the mitochondrial membrane potential, leading to uncoupled (no ATP generation) substrate flux through the mitochondria, promoting an increase in glycolysis in an attempt to maintain intracellular ATP levels. Raised BACE1 activity also had no effect on SH-SY5Y mitochondrial reserve capacity (Figures 4E,F). The results from these assays show that chronic elevation of BACE1 protein expression and activity does not impair mitochondrial electron transfer function. Consequently, this result strongly indicated that the BACE1 activity-driven decrease in glucose oxidation was the result of diminished substrate delivery to the mitochondria.

### Raised BACE1 activity lesions key metabolic pathways in oxidative glucose metabolism

**Figure 5:**
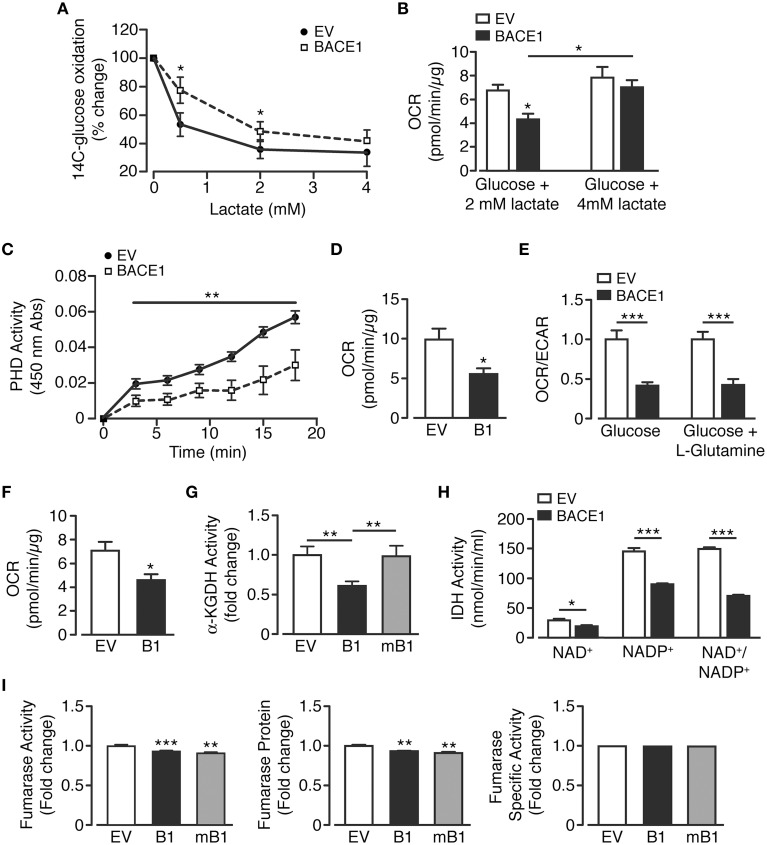
**BACE1 overexpression induces specific mitochondrial TCA cycle enzyme lesions**. **(A,B)** SH-SY5Y cells overexpressing BACE1 exhibit reduced lactate usage at levels ≤ 2mM compared to EV cells as demonstrated by **(A)** reduced repression of ^14^C-glucose oxidation (*n* = 6) and **(B)** direct OCR when lactate provided as sole substrate (*n* = 6–11). **(C)** Pyruvate dehydrogenase activity (PDH) is reduced in BACE1 overexpressing, compared to EV, cells (*n* = 5). **(D)** OCR when EV and BACE1 cells provided 2.5 mM pyruvate as sole substrate (*n* = 5). **(E)** OCR of EV and BACE1 SH-SY5Y cells when provided with 2.5 mM glucose alone or 2.5 mM glucose + 4 mM glutamine (*n* = 4–10). **(F)** direct OCR when 4 mM glutamine provided as sole substrate (*n* = 8). **(G)** BACE1, but not mBACE1, overexpression, reduced the enzyme activity of α-KGDH, compared to EV cells (*n* = 6). **(H)** IDH activity (NAD^+^ and NADP^+^ isoforms) is reduced by BACE1 overexpression compared to EV cells (*n* = 4). **(I)** BACE1 or mBACE1 overexpression has no effect on fumarase enzyme activity, protein levels or specific activity compared to EV cells (*n* = 7). Values are means ± SEM. ^*^p < 0.05; ^**^p < 0.01; ^***^p < 0.001.

There are three key control points involved in the regulation of neuronal metabolism, these are the generation of glucose-6-phosphate, pyruvate and acetyl CoA. In neurons, pyruvate is predominantly produced directly from metabolism of glucose or indirectly, through the provision of extracellular lactate via the astrocyte-neuron lactate shuttle (Magistretti and Pellerin, 1999; Itoh et al., 2003). Therefore, in an attempt to better understand the changes in cellular metabolism induced by increased BACE1 activity, lactate utilization by SH-SY5Y cells was assessed. SH-SY5Y_B1_ cells displayed significantly reduced lactate consumption as assessed by the ability of lactate to repress ^14^C-labeled glucose oxidation (Figure 5A). This deficit could also be observed as a reduction in OCR when cells were provided physiological concentrations (0.5 or 2 mM; Phypers and Pierce, 2006) of lactate as sole substrate, although the reduction in lactate metabolism was overcome by the presentation of a higher (4 mM) lactate concentration (Figures 5A,B). As the oxidation of both glucose and lactate was diminished, we hypothesized that raised BACE1 activity resulted in impairment of the pyruvate dehydrogenase complex activity (PDH). To address this we used an indirect PDH activity assay and demonstrated that SH-SY5Y_B1_ cells displayed a marked reduction of PDH activity (Figure 5C). Consistent with this outcome, SH-SY5Y_B1_cells displayed reduced OCR when provided with pyruvate (2.5 mM) as the sole substrate (Figure 5D).

In response to cellular injury or stress, compensatory metabolic strategies are employed in an attempt to bypass reduced glucose utilization associated with decreased PDH activity and obviate impaired mitochondrial TCA function. For example, glutamine metabolism is diverted from an oxidative to reductive route (Wise et al., 2011; Mullen et al., 2012), causing increased conversion to glutamate and, through glutamate dehydrogenase, raised levels of α-ketoglutarate. This additional source of α-ketoglutarate should supplement ATP generation through the TCA cycle as it bypasses the block at PDH. Consequently, we examined whether such an alternative route for TCA substrate replenishment was capable of recovering oxidative metabolism. Thus, OCR and ECAR were measured when SH-SY5Y_EV_ and SH-SY5Y_B1_ cells were provided glucose (2.5 mM) alone or glucose (2.5 mM) + glutamine (4 mM). However, the presence of glutamine did not affect the attenuation of OCR or enhancement of ECAR in SH-SY5Y_B1_ cells (Figure 5E). Moreover, OCR was reduced in SH-SY5Y_B1_ cells when glutamine was provided as the exclusive substrate (Figure 5F). These data indicate that either glutamine does not play a significant role in SH-SY5Y basal metabolism and/or that raised BACE1 activity blunts additional TCA enzymes, such as α-ketoglutarate dehydrogenase (α-KGDH), which converts α-ketoglutarate to succinyl-CoA. Indeed, SH-SY5Y_B1_ cells showed a large, BACE1 secretase activity-dependent, reduction in α-KGDH activity, which is a rate controlling step of the TCA cycle (Figure 5G).

Because, PDH and α-KGDH catalyze decarboxylation reactions involved in mitochondrial bioenergetics, we assayed the activity of the third enzyme utilizing this process, isocitrate dehydrogenase (IDH). Three isoforms of IDH are present in cells, IDH3 in the mitochondrial matrix, which reduces NAD^+^ to NADH, and IDH1 (cytoplasmic and peroxisomal) and IDH2 (mitochondrial) reducing NADP^+^ to NADPH. Consequently, specific isoform function was determined by differential supplementation (NAD^+^ or NADP^+^) of the reaction mixes. In agreement with the results for the decarboxylation enzymes above, raised BACE1 activity resulted in depression of NAD^+^- and NADP^+^-dependent IHD activity (Figure 5H). However, impairment of TCA cycle enzyme activities by increased BACE1 activity was not universal. For example, although fumarase activity was reduced slightly in SH-SY5Y_B1_ cells, this was matched in SH-SY5Y_mB1_ cells, with the protein levels of fumarase also decreased in both these cell lines, resulting in unaltered specific activity of fumarase by raised BACE1 in SH-SY5Y cells (Figure 5I). Thus, manipulation of APP cleavage toward amyloidogenic processing by increased BACE1 activity in SH-SY5Y cells reduces the overall catabolic capacity of the TCA cycle through specific enzyme lesions rather than a wholesale down-regulation.

### Rescue of glucose oxidation in SH-SY5Y_B1_ cells

**Figure 6:**
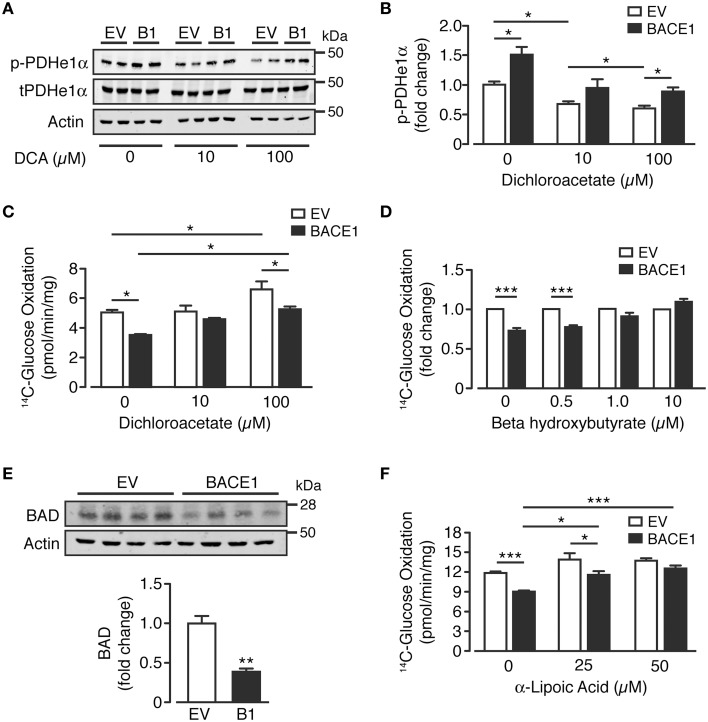
**Reversal of BACE1 mediated impaired glucose oxidation in SH-SY5Y cells**. **(A,B)** Representative immunoblots **(A)** of phosphorylated PDHe1α (p-PDHe1α) subunit, total PDHe1α and actin loading control in control (EV) and BACE1 overexpressing cells in the absence and presence of 10 μM or 100 μM dichloroacetate (DCA), with quantification of the immunoblot data shown in **(B)** (*n* = 4). **(C)** DCA partially reverses BACE1 mediated impairment of glucose oxidation, but also increases OCR of EV cells (*n* = 5). **(D)** reduced OCR associated with BACE1 cells is recovered to EV control levels by addition of 1 μM and 10 μM beta hydroxybutyrate (*n* = 4–6). **(E)** Representative immunoblots of BCL-2-associated agonist of cell death (BAD) and actin loading control with quantification of the immunoblot data (*n* = 4). **(F)** BACE1-mediated reduction in OCR is recovered to EV control levels by addition of 50 μM α-lipoic acid (*n* = 6). Values are means ± SEM. ^*^p < 0.05; ^**^p < 0.01; ^***^p < 0.001.

As glutamine supplementation was unable to recover BACE1-mediated inhibition of glucose oxidation, we focused on PDH as a potential target for pharmacological or alternative nutrient interventions. A key regulator of PDH activity is by phosphorylation of its E1α subunit (Patel et al., 2014), which is predominantly controlled by the inhibitory effect of pyruvate dehydrogenase kinases (PDKs) vs. activation through de-phosphorylation by pyruvate dehydrogenase phosphatase (PDP). Dichloroacetate (DCA) is a PDK1 inhibitor, which enhances oxidative phosphorylation in the brain (Itoh et al., 2003) and has been promoted clinically for its anti-neoplastic effects (Kankotia and Stacpoole, 2014). Consistent with PDH inhibition, SH-SY5Y_B1_ cells exhibited increased phosphorylation of the e1α subunit at serine 293 compared to SH-SY5Y_EV_ cells (Figure 6A). Treatment with DCA (10 and 100 μM) resulted in decreased levels of Ser^293^ e1α phosphorylation of both SH-SY5Y cell types, although Ser^293^ e1α phosphorylation remained significantly higher in SH-SY5Y_B1_ cells (Figures 6A,B). In agreement with the reduction in e1α phosphorylation, incubation of SH-SY5Y_EV_ cells with DCA (100 μM) increased the glucose oxidation rate (Figure 6C). However, although glucose oxidation of SH-SY5Y_B1_ cells was also enhanced by DCA treatment, this remained significantly lower than that of the SH-SY5Y_EV_ cells, mirroring e1α phosphorylation status.

An alternative means of bypassing PDH is through the application of ketones, such as beta-hydroxybutyrate (BHB), which is metabolized to acetyl-CoA and enters the TCA cycle at oxaloacetate. The presence of BHB (0.5–10 μM) recovered the relative deficit in glucose oxidation of SH-SY5Y_B1_ cells, compared to SH-SY5Y_EV_ cells, in a concentration-dependent manner (Figure 6D). Previous studies (Danial, 2008; Giménez-Cassina et al., 2012) have indicated that diminished mitochondrial consumption of glucose in neurons and the ability to switch substrate preference to ketones is dependent on the presence and activity of BAD (BCL-2-associated agonist of cell death), a member of the BCL-2 gene family member of apoptotic control proteins, with increased mitochondrial usage of BHB in *Bad*^−∕−^ cortical neurons. In agreement with this model, we find that the increased ability of SH-SY5Y_B1_ cells to utilize BHB over SH-SY5Y_EV_ cells and enhance glucose oxidation, is associated with a large reduction of BAD protein expression (Figure 6E). Finally we also investigated the ability of the naturally occurring enzyme co-factor, α-lipoic acid to modify glucose oxidation of SH-SY5Y_B1_ cells as previous studies have shown this compound to up-regulate mitochondrial bioenergetics and promote glucose uptake (Packer and Cadenas, 2011; Sancheti et al., 2013). Indeed PDH and α-KGDH protein complexes require α-lipoic acid as a co-factor for their acyl transferase activity. Supplementation of the growth media with α-lipoic acid (25 or 50 μM for 48 h) resulted in a robust concentration-dependent attenuation of the impaired glucose oxidation rate displayed by SH-SY5Y_B1_, compared to SH-SY5Y_EV_ cells (Figure 6F). Taken together, these results demonstrate the applicability of alternative nutrient (BHB) or co-factor (α–lipoic acid) supplementation to alleviate or by-pass the impaired glucose metabolism elicited by raised BACE1 activity in SH-SY5Y cells.

## Discussion

In this study we demonstrate that raised BACE1 activity by overexpression in SH-SY5Y cells, and subsequent modification of APP metabolism, induces a shift from the physiologically predominant alpha-secretase cleavage pathway to the amyloidogenic beta-secretase cleavage pathway, which results in decreased glucose metabolism. This bioenergetic impairment was dependent upon the secretase activity of BACE1 and produced a fundamental shift in the cellular metabolic profile, characterized by reduced glucose oxidation in association with a compensatory increase in glycolysis, in an attempt to maintain ATP production.

Decreased brain glucose metabolism is an invariant pathophysiological event occurring in AD progression, and it has been hypothesized that this may occur years, and even decades prior to symptom presentation (Mosconi et al., 2009a,b; Bateman et al., 2012; Jack et al., 2013c). Impaired glucose metabolism has also been noted in central tissues taken from AD animal models (von Kienlin et al., 2005; Sancheti et al., 2013; Nilsen et al., 2014). Furthermore, despite the observation that reduced glucose metabolism is predictive of later cognitive decline and AD symptom presentation, relatively little is known about the cellular mechanisms underlying these changes. Interestingly, the metabolic shift we have observed in favor of aerobic glycolysis under conditions of increased BACE1 activity mirrors early and predictive changes occurring in the brains of people who later develop AD. The brain areas that display this profile overlap with regions showing the greatest prevalence of Aβ pathology, future susceptibility to cell death and are predictive of cognitive decline (Vaishnavi et al., 2010; Vlassenko et al., 2010).

The mechanisms underlying glucose hypometabolism in AD are not well-understood. However, mitochondrial dysfunction has been widely reported in clinical and experimental AD studies (Lustbader et al., 2004; Beal, 2005; Bubber et al., 2005) and Aβ has been reported to accumulate in the mitochondria of AD patients and transgenic AD mouse models prior to amyloid deposition (Hirai et al., 2001; Devi et al., 2006; Manczak et al., 2006; Du et al., 2008). Although there are numerous reports of impaired electron transfer in mitochondria, for example via diminished activity in complexes I to V in AD subjects (Parker et al., 1994; Maurer et al., 2000; Kim et al., 2001; Bosetti et al., 2002), and diminished complex III and IV activity in a transgenic AD mouse model (Mucke et al., 2000) we found no effect of raised BACE1 activity on mitochondrial electron transfer function in SH-SY5Y cells. It may be that additional injurious processes, such as increased oxidative and/or inflammatory stress are required to act concurrently with raised BACE1 activity and increased levels of Aβ to elicit this outcome.

Thus the driving force for decreased glucose oxidation in SH-SY5Y_B1_ cells appears to be through reduced substrate delivery to mitochondria by a BACE1 activity-dependent impairment of specific TCA cycle enzymes; PDH, αKGDH and IDH each of which utilize decarboxylation reactions. Previous studies on post-mortem AD brains indicate reduced levels and/or activity of these key enzymes: PDH, αKGDH and IDH (Sorbi et al., 1983; Butterworth and Besnard, 1990; Mastrogiacoma et al., 1996; Gibson et al., 2000; Ko et al., 2001; Bubber et al., 2005), with PDH and αKGDH exhibiting the largest decreases in activity. Furthermore, Aβ peptides have been demonstrated to inhibit PDH and αKGDH directly (Shearman et al., 1994; Casley et al., 2002a,b). In contrast we find no change in the activity of the TCA enzyme fumarase, the activity of which is also unaltered in AD brains (Bubber et al., 2005). Our results add to these findings by implicating raised BACE1 activity and manipulation of APP processing as central to these enzyme deficits and metabolic adaptations at the cellular level, indicating a key role for BACE1 and its up-regulation in response to oxidative and inflammatory stress, which are associated with the very early stages of AD (Nunomura et al., 2001; Zhang et al., 2007; Guglielmotto et al., 2009). Indeed, these results show that raised BACE1 activity effectively phenocopies some of the earliest changes in glucose metabolism seen in the brain during the progression toward AD.

In situations of diminished glucose utilization, there are a number of strategies that may be engaged in an attempt to bypass this block in metabolism. For example, in brain ischemic-reperfusion injury there is also decline in glucose oxidation and PDH activity (Bogaert et al., 1994; Zaidan et al., 1998). Application of glutamine has been demonstrated to offer some protection against ischemic-reperfusion injury in peripheral tissues by providing an alternative energy source for the cell to maintain ATP content (Arsenian, 1998; Wischmeyer et al., 2003). In addition to providing a source of carbon for neurotransmitter production, glutamine can also feed into the TCA cycle and be metabolized to increase levels of α-ketoglutarate and succinate. However, our data demonstrate that glutamine supplementation is unable to restore oxidative glucose metabolism in cells overexpressing BACE1. Furthermore, SH-SY5Y_B1_ cells exhibited diminished oxidation of glutamine when applied as sole substrate, in line with the reduced activity of α-KGDH observed in these cells.

The inhibition of PDH activity in SH-SY5Y_B1_ cells was associated with increased phosphorylation of the e1α subunit at Ser^293^, which is the main inhibitory site for PDH activity (Jha et al., 2012). Although, PDK1 inhibition with dichloroacetate reduced e1α phosphorylation levels, this was also observed in SH-SY5Y_EV_ cells, in conjunction with a maintained relative lower glucose oxidation in SH-SY5Y_B1_ cells. This outcome may be owing to the presence of BACE1 activity-sensitive DCA-resistant PDK isoenzyme, or PDP, or an alternative kinase that phosphorylates e1α at Ser^293^ (e.g. GSK3). In contrast, application of the ketone, BHB, to SH-SY5Y_B1_ cells demonstrates complete recovery of glucose oxidation, relative to SH-SY5Y_EV_ cells, presumably by directly increasing the availability of acetyl CoA to the TCA cycle, thus circumventing PDH. The enhanced ability to utilize ketone bodies to aid glucose oxidation in SH-SY5Y_B1_ cells may result from their reduced BAD levels, compared to SH-SY5Y_EV_ cells. This finding agrees with the work of Nikita Danial's group showing that neurons derived from BAD knock out animals display an augmented bioenergetic profile in the presence of ketone bodies (Giménez-Cassina et al., 2012).

α-lipoic acid is synthesized in mitochondria and is a necessary cofactor for PDH and α-KGDH activity. Our finding that exogenously applied α-lipoic acid also increases glucose oxidation in SH-SY5Y_B1_, but not SH-SY5Y_EV_ cells suggests that raised BACE1 activity either reduces mitochondrial α-lipoic acid levels or lessens the ability of α-lipoic acid to enhance PDH and α-KGDH activity. The latter mechanism may be favored as there is evidence that excess α-lipoic acid provides protection to neuronal cells from the deleterious effects of exogenously applied Aβ peptides on PDH activity (Bielarczyk et al., 2006) and Aβ-induced toxicity (Zhang et al., 2001). Regarding the potential to reverse the metabolic deficits associated with age-dependent cognitive decline and early-stage or mild AD, the data presented herein demonstrate the capacity of α-lipoic acid and BHB to attenuate impaired glucose oxidation at a cellular level in face of chronically raised BACE1 activity and increased levels of Aβ peptides. BHB and α-lipoic acid have been demonstrated to exhibit some positive effects on cognitive decline in elderly dementia and mild AD patients (Hager et al., 2001; Reger et al., 2004; Costantini et al., 2008; Henderson et al., 2009; Henderson and Poirier, 2011).

Taken together, our findings are supportive for the rationale of targeting these TCA enzyme deficits in early cognitive impairment and AD. However, so far clinical trials based on raising circulating levels of ketones or giving α-lipoic acid as a dietary supplement have provided mixed outcomes and further trials examining α-lipoic acid are currently in progress. Perhaps a future strategy for AD management and treatment may be through the combination of this neutraceutical approach with new therapeutics, such as BACE1 inhibitors, currently in clinical trials (Vassar et al., 2014).

## Author contributions

JF and DH performed experiments and analyzed data. JF, DH, and MA contributed to the conception and design of experiments, interpretation of data and drafting and revising the manuscript. MA supervised the study and JF and MA wrote the manuscript. All authors approved the final version. MA is the guarantor of this work.

### Conflict of interest statement

The authors declare that the research was conducted in the absence of any commercial or financial relationships that could be construed as a potential conflict of interest.
